# Construction of a potentially functional lncRNA-miRNA-mRNA network in sepsis by bioinformatics analysis

**DOI:** 10.3389/fgene.2022.1031589

**Published:** 2022-11-15

**Authors:** Li-ming Zheng, Jun-qiu Ye, Heng-fei Li, Quan Liu

**Affiliations:** ^1^ College of Acupuncture and Orthopedics, Hubei University of Chinese Medicine, Wuhan, China; ^2^ Department of Infection, Hubei Provincial Hospital of Traditional Chinese Medicine, Affiliated to Hubei University of Chinese Medicine, Wuhan, China; ^3^ Hubei Provincial Academy of Traditional Chinese Medicine, Wuhan, China; ^4^ Department of Pulmonary Disease, Hubei Provincial Hospital of Traditional Chinese Medicine, Affiliated to Hubei University of Chinese Medicine, Wuhan, China

**Keywords:** bioinformatics analysis, competing endogenous RNA network (ceRNA network), mRNA-miRNA-lncRNA, sepsis, therapeutic targets

## Abstract

**Objective:** Sepsis is a common disease in internal medicine, with a high incidence and dangerous condition. Due to the limited understanding of its pathogenesis, the prognosis is poor. The goal of this project is to screen potential biomarkers for the diagnosis of sepsis and to identify competitive endogenous RNA (ceRNA) networks associated with sepsis.

**Methods:** The expression profiles of long non-coding RNAs (lncRNAs), microRNAs (miRNAs) and messenger RNAs (mRNAs) were derived from the Gene Expression Omnibus (GEO) dataset. The differentially expressed lncRNAs (DElncRNAs), miRNAs (DEmiRNAs) and mRNAs (DEmRNAs) were screened by bioinformatics analysis. DEmRNAs were analyzed by protein-protein interaction (PPI) network analysis, transcription factor enrichment analysis, Gene Ontology (GO), Kyoto Encyclopedia of Genes and Genomes (KEGG) pathway analysis and Gene Set Enrichment Analysis (GSEA). After the prediction of the relevant database, the competitive ceRNA network is built in Cytoscape. The gene-drug interaction was predicted by DGIgb. Finally, quantitative real-time polymerase chain reaction (qRT-PCR) was used to confirm five lncRNAs from the ceRNA network.

**Results:** Through Venn diagram analysis, we found that 57 DElncRNAs, 6 DEmiRNAs and 317 DEmRNAs expressed abnormally in patients with sepsis. GO analysis and KEGG pathway analysis showed that 789 GO terms and 36 KEGG pathways were enriched. Through intersection analysis and data mining, 5 key KEGG pathways and related core genes were revealed by GSEA. The PPI network consists of 247 nodes and 1,163 edges, and 50 hub genes are screened by the MCODE plug-in. In addition, there are 5 DElncRNAs, 6 DEmiRNAs and 28 DEmRNAs in the ceRNA network. Drug action analysis showed that 7 genes were predicted to be molecular targets of drugs. Five lncRNAs in ceRNA network are verified by qRT-PCR, and the results showed that the relative expression of five lncRNAs was significantly different between sepsis patients and healthy control subjects.

**Conclusion:** A sepsis-specific ceRNA network has been effectively created, which is helpful to understand the interaction between lncRNAs, miRNAs and mRNAs. We discovered prospective sepsis peripheral blood indicators and proposed potential treatment medicines, providing new insights into the progression and development of sepsis.

## Introduction

Sepsis is an infection-related condition characterized by a systemic inflammatory reaction, often resulting in extensive tissue damage, such as the development of multi-organ dysfunction, systemic hypotension, renal hypoperfusion and renal ischemia-reperfusion (Singer et al., 2016). About 18 million new cases of sepsis are diagnosed worldwide each year, and the number is increasing year by year (Zhou et al., 2013). According to relevant epidemiological surveys, sepsis’ morbidity and mortality rate has surpassed that of myocardial infarction, and approximately 14,000 people die from sepsis complications every day, making it the leading cause of death among non-cardiac patients in intensive care units (ICUs), posing a great threat to human health (Keppler et al., 2018; Pellegrini et al., 2021).

Although sepsis was treated appropriately, the overall clinical outcome was unsatisfactory. In addition, available studies suggest that early diagnosis is quite difficult due to several complications and the lack of effective predictive techniques (Novosad et al., 2016). Therefore, new biomarkers linked with sepsis are urgently needed for early diagnosis, monitoring, and therapeutic intervention in sepsis. Many researchers have sought to discover novel sepsis biomarkers. Previous studies suggested that triggering receptor expressed on myeloid cells-1 (TREM-1), IL-27, neutrophil CD64, preprotease, and cell-free plasma DNA (cfDNA) were new promising biomarkers for sepsis diagnosis and therapy (Sandquist and Wong, 2014). Newly discovered biomarker classes, including microRNAs (miRNAs), long non-coding RNAs (lncRNAs), and the human microbiome, are also of widespread interest (Kim and Choi, 2020). Despite the increased number of putative biomarkers, these efforts have yet to provide satisfying findings, necessitating more validation.

MiRNAs are single-stranded ncRNA molecules with a length of 21–24 nucleotides that bind to complementary sequences in the 30 untranslated regions (UTRs) of target messenger RNAs (mRNAs), causing mRNA degradation or inhibition (Sandquist and Wong, 2014). Mirna-186 has been shown to ameliorate renal injury caused by sepsis *via* the PTEN/PI3K/Akt/p53 pathway (Li et al., 2020). LncRNAs are non-protein-coding RNAs with a length of more than 200 nucleotides that play key roles in biological processes, participating in post-transcriptional regulation, cell-cell signaling, and protein metamorphosis regulation (Mattick and Rinn, 2015). In animal experiments, researchers have found that lncRNA-NEAT1 gene knockout can inhibit TLR2/NF-κB signaling pathway improves myocardial injury induced by sepsis (Wang et al., 2019). Salmena et al. (Salmena et al., 2011) suggested that molecules may have a regulatory function in competing for endogenous RNAs (ceRNA) by competing with the same miRNA response element, and the ceRNA hypothesis indicates that lncRNAs can sponge-bind and inactivate miRNAs, eventually lowering mRNA degradation or suppressing mRNA translation and thereby influencing protein coding. In the background of ceRNA network, there are few comprehensive analyses on the association between ceRNA network and sepsis. The identification of the interaction between ceRNA network and sepsis may provide important enlightenment for us to better understand the pathogenesis of sepsis.

**FIGURE 1:**
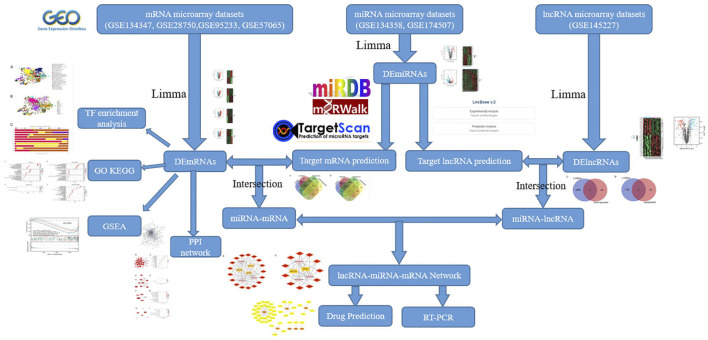
Flowchart of the present study.

In this work, we utilized bioinformatics approaches to detect differentially expressed lncRNAs (DElncRNAs), miRNAs (DEmiRNAs), and mRNAs (DEmRNAs) in the sepsis gene expression datasets from the National Center for Biotechnology Information Gene Expression Omnibus (NCBI GEO) database. We created a protein protein interaction (PPI) network and recognized hub genes. Then, in order to further investigated the key biological functions of DEmRNAs, we performed transcription factor (TF) enrichment, Gene Ontology (GO) enrichment, Kyoto Encyclopedia of Genes and Genomes (KEGG) pathway analysis and Gene set enrichment analysis (GSEA) on DEmRNAs. Next, we built a lncRNA-miRNA-mRNA network based on the ceRNA theory to define the functional lncRNAs in sepsis, screen critical lncRNAs substantially linked to the disease, predict their molecular regulation mechanisms, and find new targets for diagnostic and therapy. Finally, using qRT-PCR, key lncRNAs were molecularly confirmed (Figure 1). To the best of our knowledge, this is the first research to analyze the differential expression profiles of the ceRNA network in sepsis using bioinformatics tools.

## Materials and methods

### GEO dataset collection

Seven expression profile datasets (GSE145227, GSE134358, GSE174507, GSE134347, GSE28750, GSE95233, GSE57065) were downloaded from the GEO database. GSE145227 is a lncRNAs expression profile, involving 22 plasma samples (10 pediatric sepsis patients vs. 12 healthy controls) and was detected on the platform of GPL23178 (Affymetrix Human Custom lncRNA Array). GSE134358 and GSE174507 are expression profiles of miRNAs. 158 ICU patients with sepsis and 82 healthy subjects were selected in GSE134358, and the detection platform was GPL21572 (Affymetrix Multispecies miRNA-4 Array). GSE174507 contained 12 sepsis patients and 6 control donors, and was associated the GPL25134 platform (Agilent-070156 Human_miRNA_V21.0_Microarray 046064). GSE134347, GSE28750, GSE95233 and GSE57065 are expression profiles of mRNAs. The GSE134347 dataset included gene expression profiling based on arrays of whole blood from 156 ICU patients with sepsis and 83 healthy subjects, and the detection platform was GPL17586 (Affymetrix Human Transcriptome Array 2.0). The GSE28750 dataset included gene expression profiling from 10 sepsis patients and 20 healthy controls, and the detection platform was GPL570 (Affymetrix Human Genome U133 Plus 2.0 Array). 51 septic shock patients and 22 healthy volunteers were included in GSE95233, septic shock patients were sampled twice after admission, and the detection platform was GPL570 (Affymetrix Human Genome U133 Plus 2.0 Array). GSE57065 included 28 ICU patients. Blood samples were collected within 30 min, 24 h and 48 h after septic shock and compared to 25 healthy volunteers, and the detection platform was GPL570 (Affymetrix Human Genome U133 Plus 2.0 Array). Table 1 shows the information collected from the datasets. Because all data had been published into the public domain, no Institutional Review Board approval was necessary for this study.

**TABLE 1:** Basic information of the 7 microarray datasets from GEO.

Type	Series	Platform	Source name	Samples (c)
lncRNA	GSE14522	GPL23178	blood	22 (12/10)
miRNA	GSE13435	GPL21572	blood	240 (82/15
miRNA	GSE17450	GPL25134	blood	18 (6/12)
mRNA	GSE13434	GPL17586	blood	239 (83/15
mRNA	GSE28750	GPL570	blood	30 (20/10)
mRNA	GSE95233	GPL570	blood	124 (22/10
mRNA	GSE57065	GPL570	blood	107 (25/82)

### Differential expression analysis

Firstly, we preprocess the original files obtained from the GEO database, and probes relating to multiple molecules are eliminated; when probes related to the same molecule are discovered, only the probe with the highest signal value is maintained. Gene analysis of differences between samples was performed using the limma package (v3.42.0) in R software (v3.6.3). The criteria for DElncRNAs were FDR (adjusted *p*-value) < 0.05 and |log_2_ fold-change|>0.75. The criteria for DEmiRNAs were FDR < 0.05 and |log_2_FC|>0.5. The criteria for DEmRNAs were FDR < 0.05 and |log_2_FC|>1. The VennDiagram R package was used to create a Venn diagram. The ComplexHeatmap package (v2.2.0) and the ggplot2 package (v3.3.3) were used to create the heatmap and volcano.

### Construction of the PPI network

The PPI network was built using the online tool STRING (https://string-db.org/) (Szklarczyk et al., 2019) and a filter condition (combined score>0.4) based on all DEmRNAs. Next, we used Cytoscape software (v3.8.2) to download the interaction data and improve the PPI network to find the important modules and hub genes (Shannon et al., 2003). The functional modules were predicted using the MCODE plug-in (v2.0.0) (Bader and Hogue, 2003), and the module with a score greater than 3.5 was chosen for KEGG pathway analyses.

### Gene ontology and Kyoto encyclopedia of genes and genomes enrichment analysis

To gain a better understanding of the DEmRNAs’ potential functional annotation and pathway enrichment, the clusterProfiler package (v3.14.3) (Yu et al., 2012) was used to perform Gene Ontology (GO) analyses, including biological process (BP), cellular component (CC), molecular function (MF), and KEGG pathway analyses, with *p* < 0.05 indicating statistically significant differences.

### Gene set enrichment analysis

GSEA was used to determine the key pathways and core genes during the development of sepsis (Subramanian et al., 2005). The default weighted enrichment method was applied for enrichment analysis. The random combination was set for 1,000 times. FDR < 0.25, *p*-value < 0.01 and |NES|> 1 were considered significant enrichment. ClusterProfiler package (v3.14.3) (Yu et al., 2012) was applied to visualize the results, which presented the dysfunctional pathways in sepsis population compared with the normal population. Ggplot2 package (v3.3.3) was used for ridge plot.

### Transcription factor enrichment analysis

We performed an enrichment analysis of transcription factor (TF) using the ChEA3 software for DEmRNAs, as these TFs were likely to be useful in further research into the mechanism of sepsis.

### Construction of the ceRNA network

The lncRNA-miRNA-mRNA network was built according to ceRNA’s hypothesis. Target DEmRNAs were predicted for DEmiRNAs using miRDB (http://www.mirdb.org/) (Wang, 2016), miRWalk (http://mirwalk.umm.uni-heidelberg.de/) (Sticht et al., 2018), and TargetScan database (http://www.targetscan.org/) (Fromm et al., 2015) respectively. The key mRNAs, we thought, were the intersection results of mRNAs predicted in the database and DEmRNAs analyzed in the R software. Then, we used DIANA-LncBase (v2) database (Agarwal et al., 2015) databases to predict miRNA-bound lncRNAs. Accordingly, the intersection of lncRNAs predicted in the database and DElncRNAs analyzed in R software is considered the important lncRNAs. Finally, all of the data was imported into Cytoscape to create a lncRNA-miRNA-mRNA network.

### Drug prediction

DGIgb (v3.0) was used to forecast drugs for mRNAs in the lncRNA-miRNA-mRNA network.

### Verification of key lncRNAs

To validate the key identified lncRNAs, we selected nine blood samples from sepsis patients (Table 2) and nine blood samples from healthy control subjects for qRT-PCR molecular validation. We extracted total RNA from the blood using the TRIpure reagent (ELK Biotechnology, EP013). M-MLV Reverse Transcriptase reagent Kit (ELK Biotechnology, EQ002) was used to synthesize cDNA according to the manufacturer’s instructions. Then, qRT-PCR was performed using the QuFast SYBR Green PCR Master Mix Kit (ELK Biotechnology, EQ001) to quantify the expression levels of lncRNAs, on a real-time PCR system (StepOnePlus; Applied Biosystems). GAPDH was used as the internal reference gene. Finally, we analyzed the data by the comparative quantitative cycle (Cq) (2-ΔΔCq) method. The research was approved by the Ethics Committee of Hubei Provincial Hospital of Traditional Chinese Medicine. All patients provided written informed consent for research on their specimens. Supplementary Table S1 shows the primer sequences used for qRT-PCR.

**TABLE 2:** Detailed clinical information of 9 sepsis patients.

Patients	Age (year)	Sex	Body mass index (kg/m^2^)	SOFA score	APACHE II score
Patient1	49	Female	22.07	11	25
Patient2	55	Female	26.44	10	28
Patient3	71	Male	22.54	12	23
Patient4	69	Female	18.45	9	26
Patient5	77	Male	20.72	8	25
Patient6	58	Male	25.65	9	24
Patient7	70	Female	19.32	10	26
Patient8	66	Male	17.78	10	27
Patient9	74	Female	21.83	8	25

SOFA: sequential organ failure assessment; APACHE: acute physiology and chronic health evaluation.

### Statistical analysis

The data were given as mean ± standard deviation (SD). Statistical analyses were operated by SPSS 26.0 (SPSS, Inc., Chicago, IL, United States) and GraphPad Prism 7.0 software. Each experiment was carried out at least three times. The student’s *t*-test was applied to analyze two sets of parameters. The statistical significance was *p* < 0.05.

## Results

### Differential expression analysis

**FIGURE 2:**
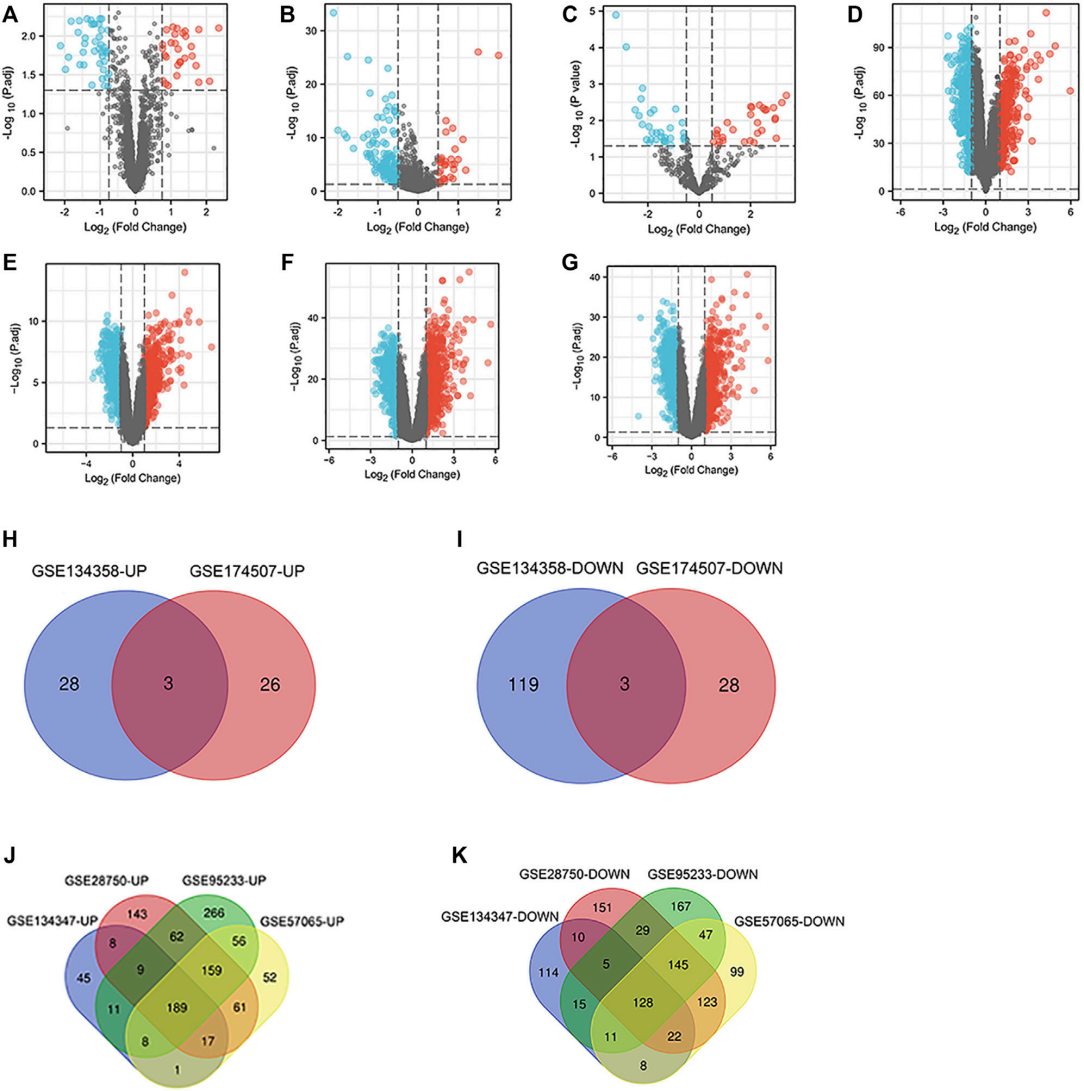
DElncRNAs, miRNAs and mRNAs between the sepsis and normal samples. **(A)** Volcano map of the GSE145227 dataset. **(B)** Volcano map of the GSE134358 dataset. **(C)** Volcano map of the GSE174507 dataset. **(D)** Volcano map of the GSE134347 dataset. **(E)** Volcano map of the GSE63492 dataset. **(F)** Volcano map of the GSE95233 dataset. **(G)** Volcano map of the GSE57065 dataset. Red spots represent upregulated genes, and blue spots represent downregulated genes in volcano maps. **(H,I)** Venn diagrams represent the intersections of upregulated and downregulated DEmiRNAs in the GSE134358 and GSE174507 datasets. **(J,K)** Venn diagrams represent the intersections of upregulated and downregulated DEmRNAs in the GSE134347, GSE63492, GSE95233 and GSE57065 datasets. DElncRNAs, differentially expressed lncRNAs; DEmiRNAs, differentially expressed miRNAs; DEmRNAs, differentially expressed mRNAs.

Seven microarray datasets from the GEO were included in this study. GSE145227 contained 57 DElncRNAs (32 downregulated and 25 upregulated) (Figure 2A). GSE134358 contained 153 DEmiRNAs (122 downregulated and 31 upregulated) (Figure 2B), and GSE174507 contained 60 DEmiRNAs (31 downregulated and 29 upregulated) (Figure 2C). Besides, GSE134347 and GSE63492 contained 601 (313 downregulated and 288 upregulated) (Figure 2D) and 1,261 (613 downregulated and 648 upregulated) DEmRNAs (Figure 2E), respectively. GSE95233 contained 1,306 DEmRNAs (546 downregulated and 760 upregulated) (Figure 2F), and GSE57065 contained 1,126 DEmRNAs (583 downregulated and 543 upregulated) (Figure 2G). Next, heatmap and volcano plot analyses were used to visualize these DEGs. Volcano plot analyses were shown in Figures 2A–G, heatmap plot analyses were shown in Supplementary Figures S1–S7. Venn diagram identified 6 (3 downregulated and 3 upregulated) common DEmiRNAs and 317 (128 downregulated and 189 upregulated) common DEmRNAs (Figures 2H–K).

### PPI network analysis

**FIGURE 3:**
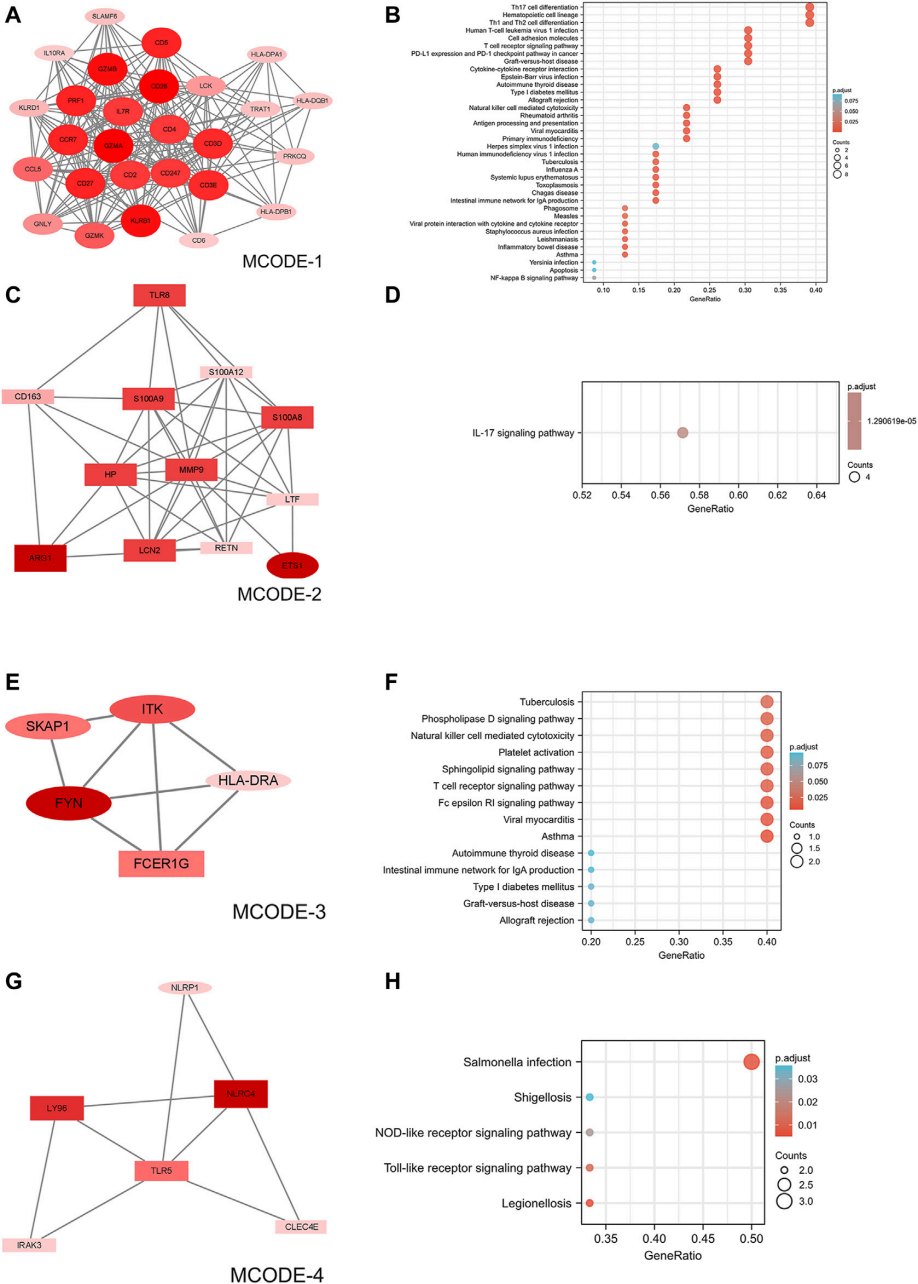
PPI network of DEmRNAs and four cluster modules extracted by MCODE. **(A)** The interaction network between proteins coded by DEmRNAs was comprised of 317 nodes and 1,163 edges. **(B,D,F,H)** Use Cytoscape plugin MCODE to filter the important modules in DEmRNAs, and then filter the final 4 important modules according to the filtering criteria. The nodes represent proteins of DEmRNAs, while each edge represents one protein–protein association. Larger and darker nodes (proteins) indicate more interactions (higher degree). Red diamonds represent the upregulated genes, and green hexagons represent the downregulated genes. Ellipses represent downregulated mRNAs and rectangles represent upregulated mRNAs. **(C,E,G,I)** Results of the 4 important modules pathway enrichment analyses. DEmRNAs, differentially expressed mRNAs.

Online tool STRING generated a PPI network of differentially expressed mRNAs with 247 nodes and 1,163 interaction connections (Supplementary Figure S8). Following MCODE’s functional module study, four clusters were proposed as functional modules (scores>3.5). In cluster 1, there were 27 downregulated mRNAs (Figure 3A); in cluster 2, there were 12 upregulated and one downregulated mRNAs (Figure 3C); in cluster 3, there were 1 upregulated and four downregulated mRNAs (Figure 2F); and in cluster 4, there were 5 upregulated and one downregulated mRNAs (Figure 3G). According to function analysis, genes in cluster 1 were predominantly related with Th17 cell differentiation, hematopoietic cell lineage, and Th1 and Th2 cell differentiation (Figure 3B); those in cluster 2 were primarily associated with IL-17 signaling pathway (Figure 3D); those in cluster 3 were primarily associated with tuberculosis, phospholipase D signaling pathway, natural killer cell mediated cytotoxicity, platelet activation, sphingolipid signaling pathway, T cell receptor signaling pathway, Fc epsilon RI signaling pathway, viral myocarditis, and asthma (Figure 3F); and those in cluster 4 were primarily associated with *Salmonella* infection (Figure 3H).

### Transcription factor enrichment analysis

**FIGURE 4:**
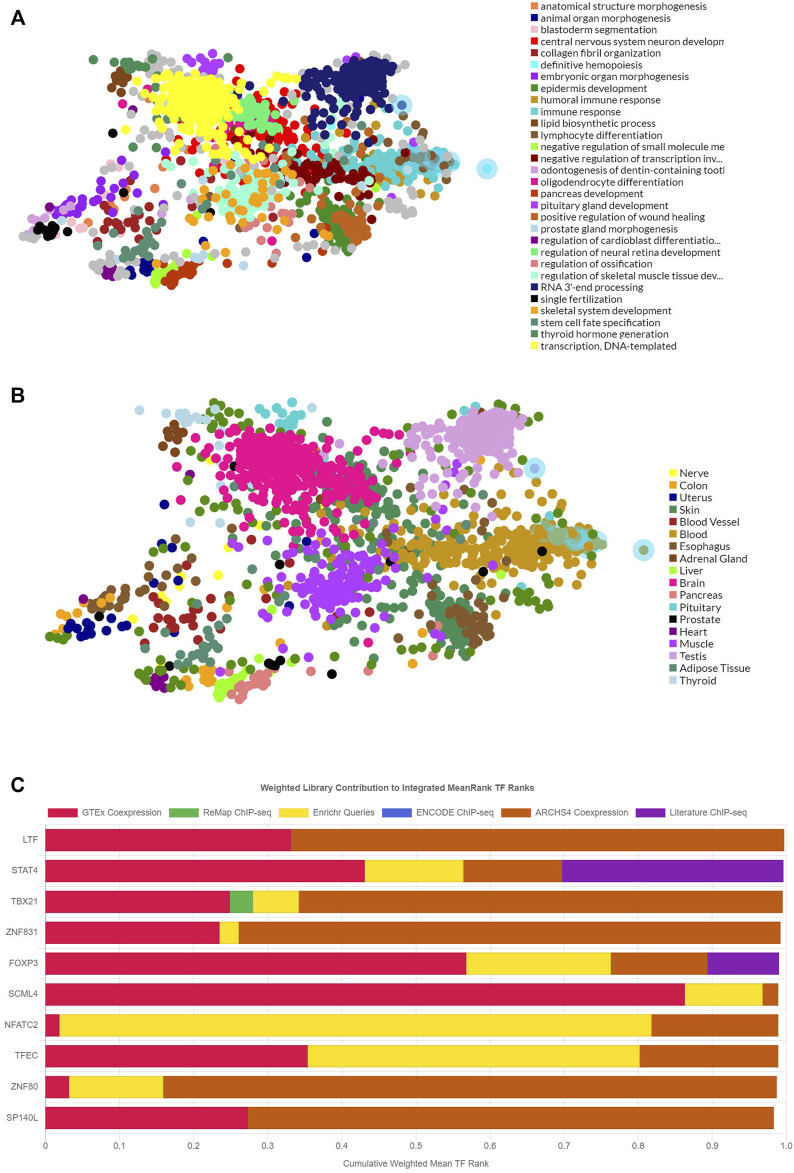
The functions, locations, and the top-ranking TF targets of the 317 DEmRNAs. **(A)** The main biological functions of the 317 DEmRNAs. **(B)** The tissue distributions of the TF targets of the 317 DEmRNAs. **(C)** The top 10 TF targets of the 317 DEmRNAs. DEmRNAs, differentially expressed mRNAs; TF: transcriptional factor.

The TF targets of common DEmRNAs were enriched by using ChEA3 so that their distribution and biological roles could be investigated further. TFs can regulate transcription and so perform a regulatory role. The results showed that the functions of the TF targets included transcription, immune response, and animal organ morphogenesis (Figure 4A). The TFs were validated that were distributed into diverse tissues, such as the brain, testis, and adipose tissue (Figure 4B). The top 10 TFs included LTF, STAT4, TABX21, ZNF831, FOXP3, SCML4, NFATC2, TFEC, ZNF80, and SP140L (Figure 4C).

### GO and KEGG analysis

**FIGURE 5:**
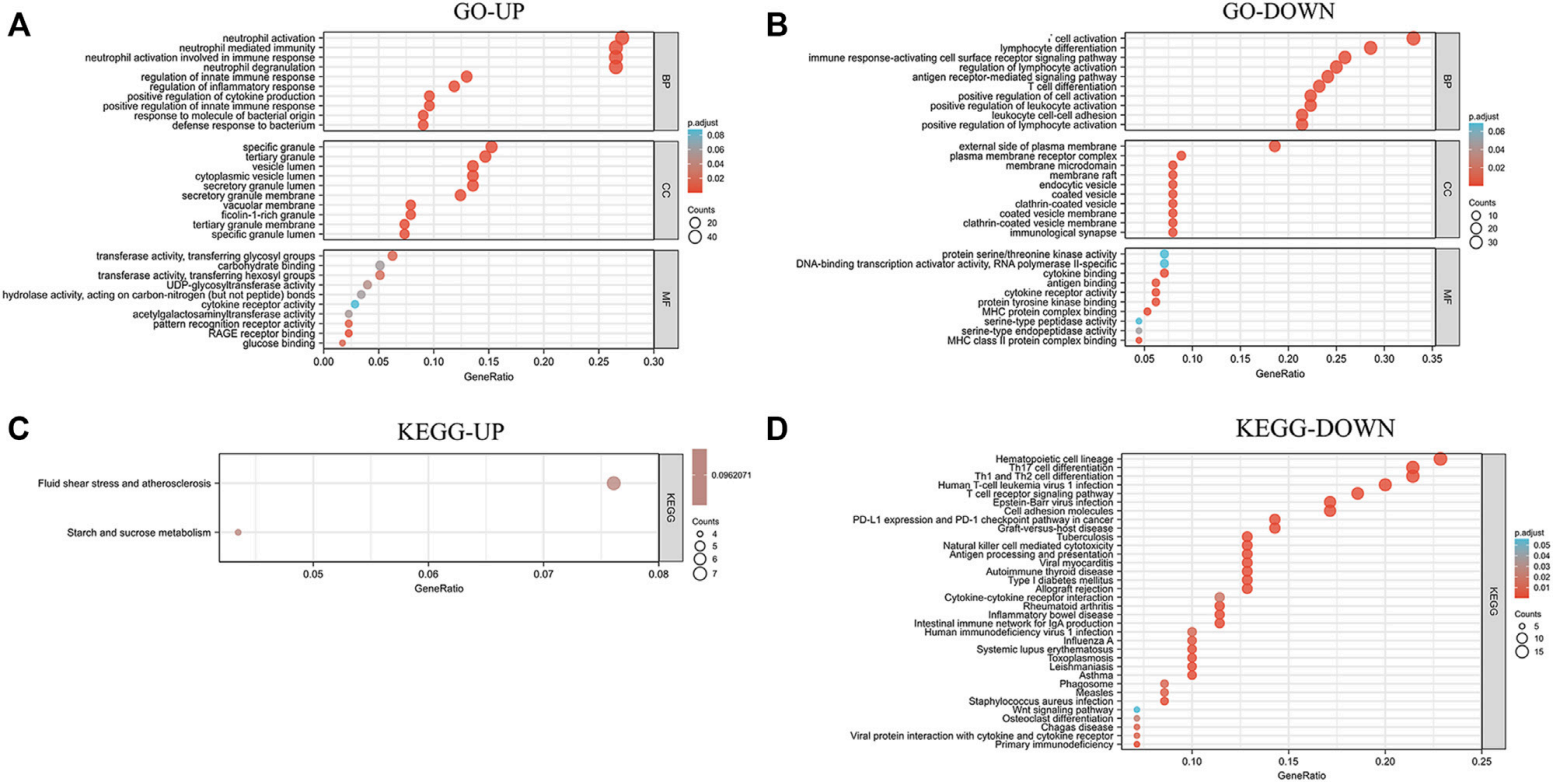
Analysis results of co-expressed genes in the GO and KEGG pathways. **(A,B)** Results of GO enrichment analysis of UP and DOWN DEmRNAs. **(C,D)** Results of KEGG pathway analysis of UP and DOWN DEmRNAs. DEmRNAs, differentially expressed mRNAs; GO, Gene Ontology; KEGG, Kyoto Encyclopedia of Genes and Genomes; UP, upregulated; DOWN, downregulated.

To further investigate the biological function of the DEmRNAs, the clusterProfiler package in R was used to perform GO and KEGG enrichment analyses. In the DEmRNAs upregulation group, 373 GO terms and 2 KEGG pathways were enriched, and in the DEmRNAs downregulation group, 416 GO terms and 34 KEGG pathways were significantly enriched. In the biological process (BP) group, upregulated DEmRNAs were primarily enriched in neutrophil activation, neutrophil degranulation, neutrophil activation involved in immune response, and neutrophil mediated immunity (Figure 5A). In contrast, downregulated DEmRNAs were mostly enriched in T cell activation, lymphocyte differentiation, and immune response-activating cell surface receptor signaling pathway (Figure 5B). In the cellular component (CC) group, upregulated DEmRNAs were primarily enriched in specific granule and tertiary granule (Figure 5A). In contrast, downregulated DEmRNAs were mostly enriched in external side of plasma membrane and plasma membrane receptor complex (Figure 5B). In the molecular function (MF) group, upregulated genes were primarily enriched in transferase activity (transferring glycosyl groups), carbohydrate binding, and transferase activity (transferring hexosyl groups) (Figure 5A), while downregulated DEmRNAs were mostly enriched in protein serine/threonine kinase activity, DNA-binding transcription activator activity (RNA polymerase II-specific), and cytokine binding (Figure 5B). Moreover, KEGG pathway analysis showed that the upregulated DEmRNAs were significantly enriched in fluid shear stress and atherosclerosis, starch and sucrose metabolism (Figure 5C). In contrast, downregulated DEmRNAs were considerably enriched in hematopoietic cell lineage, Th17 cell differentiation, and Th1 and Th2 cell differentiation (Figure 5D).

### GSEA enrichment of expression datasets

**FIGURE 6:**
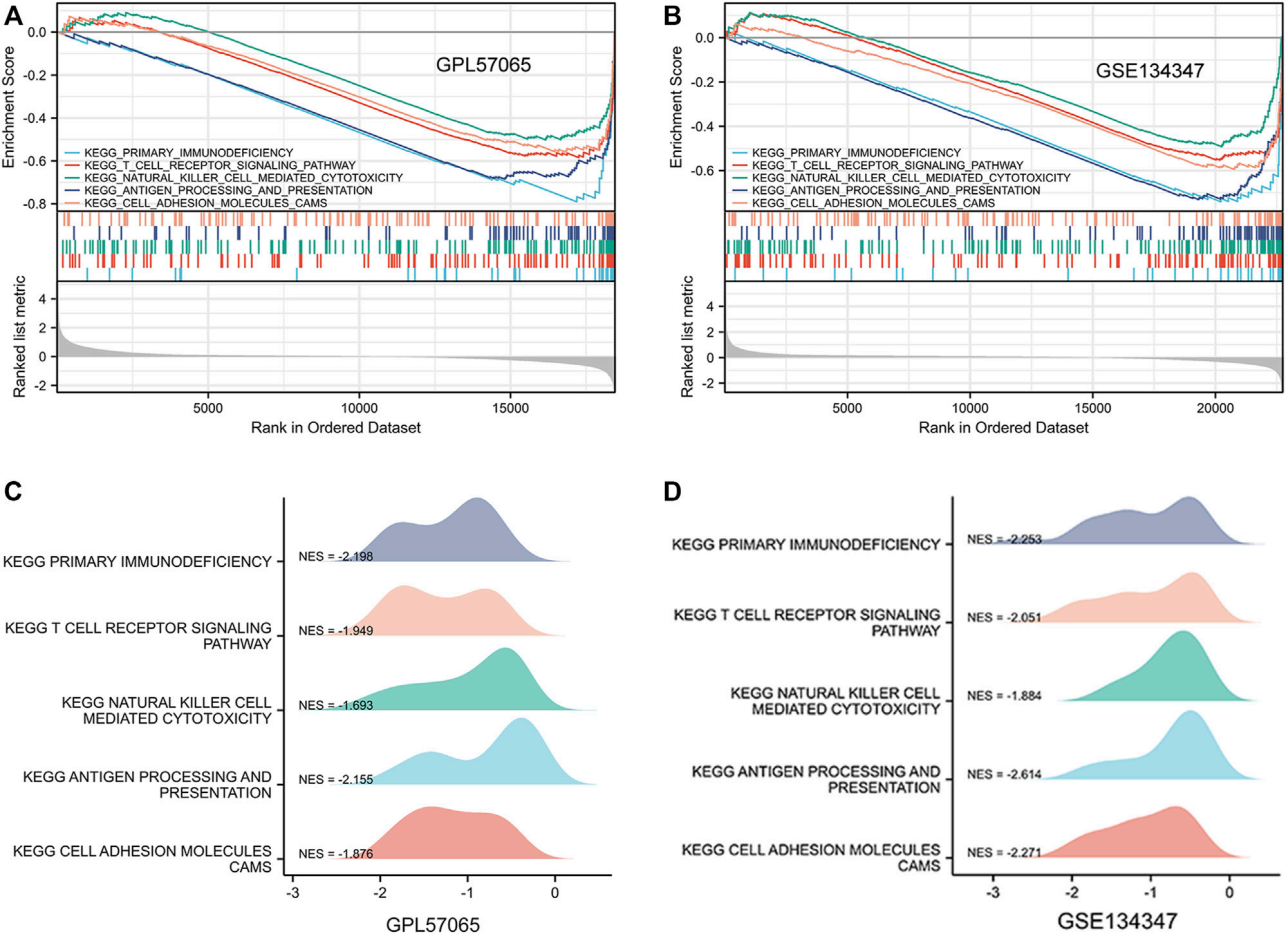
Gene set enrichment analysis (GSEA) was used to analyze the KEGG pathways enrichment in different groups. **(A)** GSEA using three data sets of GPL57065. **(B)** GSEA using GSE134347. **(C)** GPL57065 ridge plot. **(D)** GSE134347 ridge plot.

To determine the potential function of bona fide hub genes in sepsis, we performed GSEA on expression data sets from the GPL570 and GPL17586 platforms, searching for KEGG pathways enriched in high-expression samples. The results showed that five KEGG pathways were enriched after screening: primary immunodeficiency, T cell receptor signaling pathway, natural killer cell mediated cytotoxicity, antigen processing and presentation, cell adhesion molecules cams (Table 3) (Figure 6).

**TABLE 3:** The significant enriched KEGG pathways from GSEA results (*p* < 0.01, FDR < 0.25).

ID	NES		P-value		FDR	
	GPL57065	GSE134347	GPL57065	GSE134347	GPL57065	GSE134347
Primary_immunodeficiency	-2.197614	-2.2530588	0.00188	0.0017762	0.046734	0.0307762
T_cell_receptor_signaling_pathway	-1.948881	-2.0513538	0.001927	0.0016474	0.046734	0.0307762
Natural_killer_cell_mediated_cytotoxicity	-1.693193	-1.8844068	0.001996	0.0016393	0.046734	0.0307762
Antigen_processing_and_presentation	-2.155499	-2.6139374	0.002016	0.0016667	0.046734	0.0307762
Cell_adhesion_molecules_cams	-1.876137	-2.2705177	0.002028	0.0016393	0.046734	0.0307762

NES: normalized enrichment score; FDR: false discovery rate.

### Construction of the lncRNA-miRNA-mRNA

**FIGURE 7:**
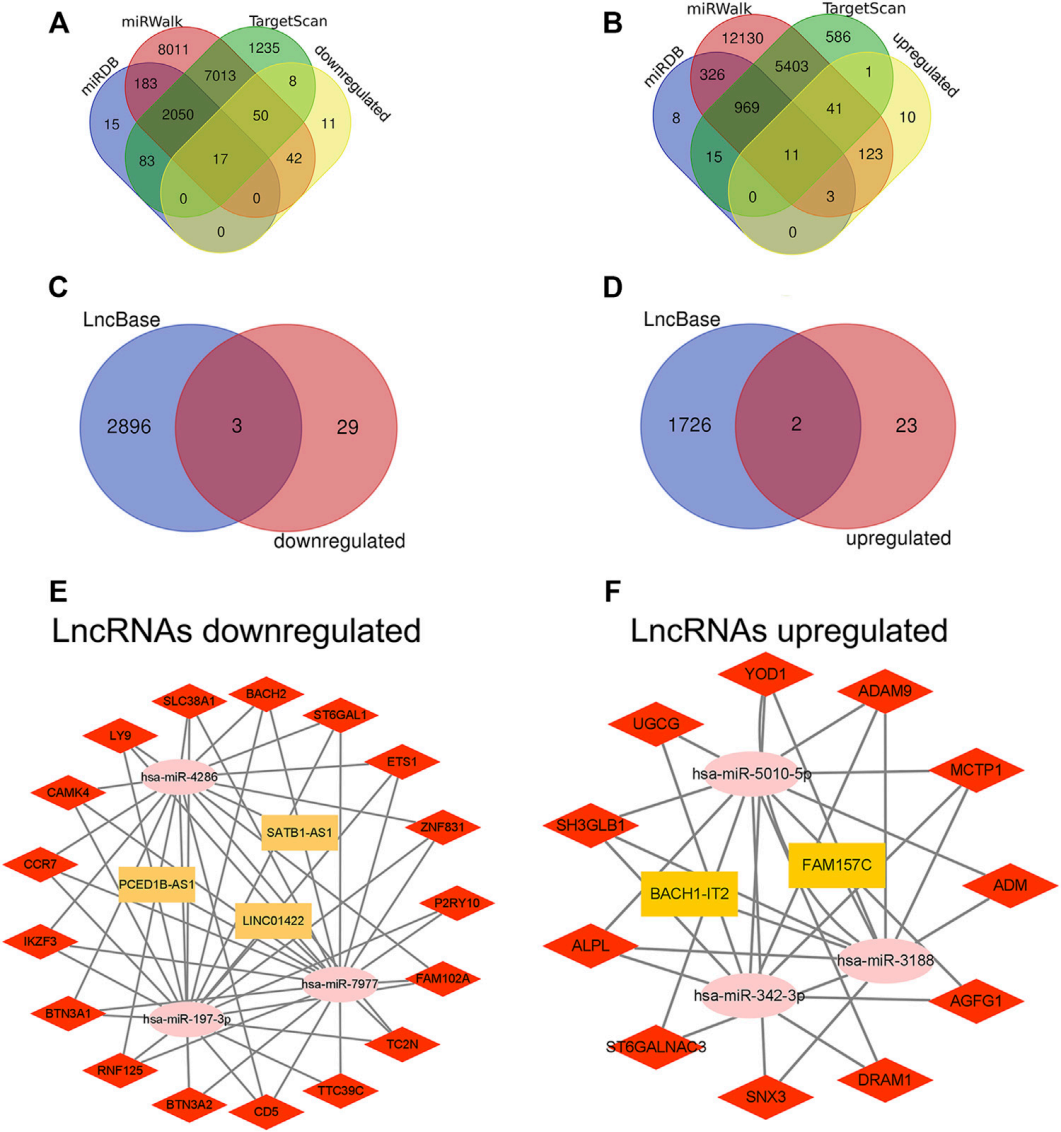
Construction of ceRNA network in sepsis. **(A,B)** Venn diagram showing the number of distinct and overlapping mRNAs among the downregulated and upregulated DEmRNAs, and the mRNAs identified with miRDB, miRWalk, and TargetScan. The overlapping areas show the downregulated and upregulated mRNAs identified by three online tools. **(C,D)** Venn diagram showing the number of distinct and overlapping lncRNAs among the downregulated and upregulated DElncRNAs, and the lncRNAs identified with LncBase. The overlapping areas show the downregulated and upregulated lncRNAs identified by Lncbase online tool. **(E,F)** Construction of a complete lncRNA-miRNA-mRNA ceRNA network according to the upregulation and downregulation of lncRNAs. The yellow rectangles represent DElncRNAs, the pink ellipses represent DEmiRNA, and the red diamonds represent DEmRNAs. DElncRNAs, differentially expressed lncRNAs; DEmiRNAs, differentially expressed miRNAs; DEmRNAs, differentially expressed mRNAs.

We used the miRDB database to predict target mRNAs of DEmiRNAs, these mRNAs were also found in the miRWalk database and TargetScan database. Then, we found 28 mRNAs (17 downregulated and 11 upregulated) by intersecting the mRNAs in the predicted mRNA database and DEmRNAs (Figures 7A,B). Furthermore, the LncBasev.2 database was utilized to predict the lncRNAs that regulate miRNAs (threshold>0.7), and the Venn diagram was used to intersect DElncRNAs with the predicted lncRNAs. The ceRNA hypothesis states that between lncRNAs and mRNAs, there is a positive regulatory interaction, but between miRNAs and mRNAs, there is a negative regulatory relationship. Finally, we identified 5 lncRNAs (3 downregulated and 2 upregulated) with substantial variations in expression during sepsis (Figures 7C,D). Using Cytoscape, we built the ceRNA network. When lncRNAs were downregulated, the network consisted of 3 downregulated lncRNA nodes, 3 upregulated miRNA nodes, 17 downregulated mRNA nodes, 4 lncRNA-miRNA pairs, and 49 miRNA-mRNA pairs (Figure 7E). When lncRNAs were upregulated, the network consisted of 2 upregulated lncRNA nodes, 3 downregulated miRNA nodes, 11 upregulated mRNA nodes, 2 lncRNA-miRNA pairs, and 27 miRNA-mRNA pairs (Figure 7F).

### Drug prediction for mRNA in lncRNA-miRNA-mRNA network

**FIGURE 8:**
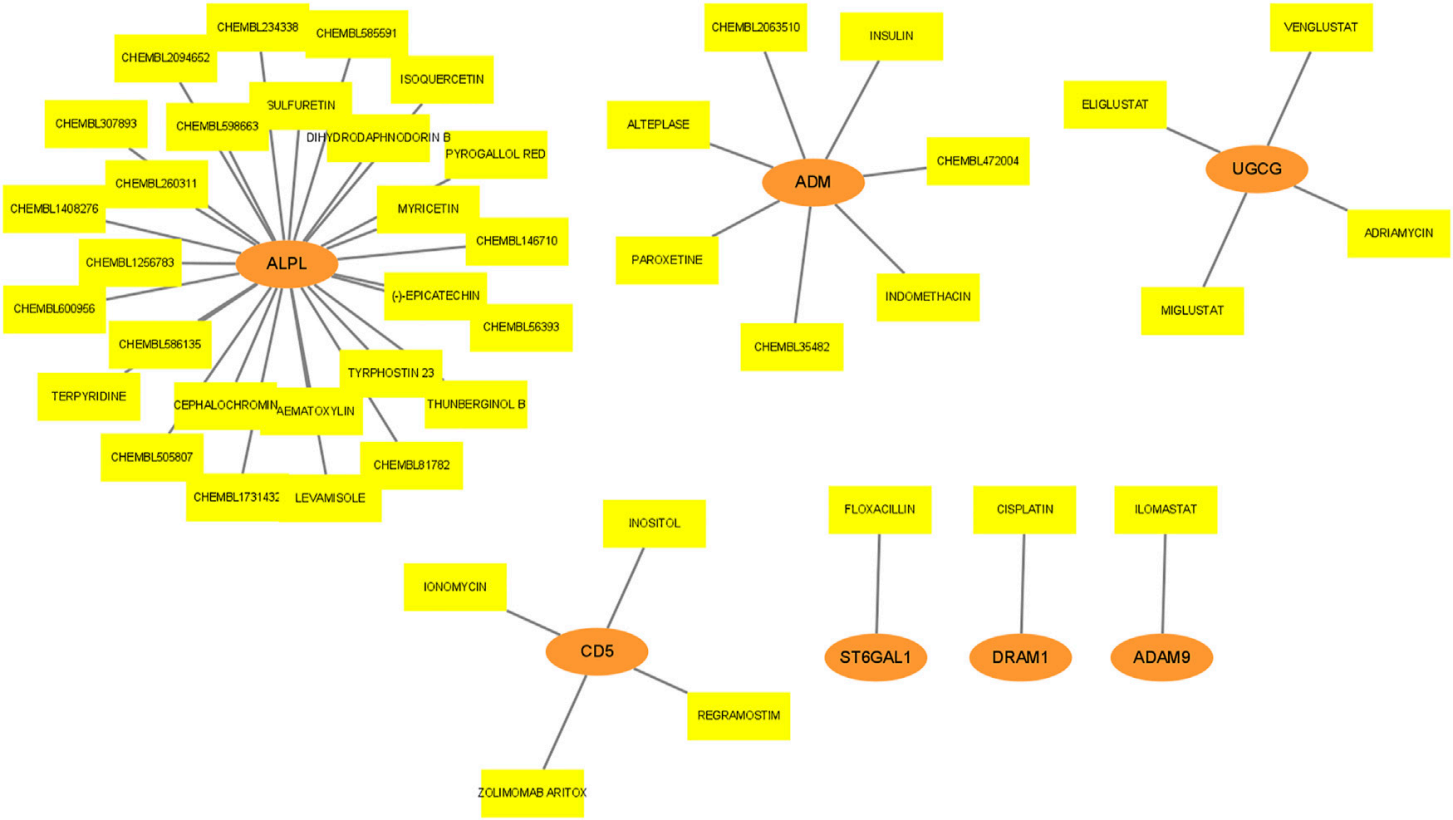
Drug prediction for mRNA in lncRNA-miRNA-mRNA network. The orange ellipses represent mRNAs, and the yellow rectangles represent drugs targeted by mRNAs.

Drug action analysis was done for twenty-eight differential mRNAs included in the lncRNA-miRNA-mRNA network, and seven genes were predicted as molecular targets of drugs, involving 45 drug-target pairs (Figure 8). We found that alkaline phosphatase, biomineralization associated (ALPL) was targeted by 27 drugs. Adrenomedullin (ADM) was targeted by 7 drugs. UDP-glucose ceramide glucosyltransferase (UGCG) and CD5 molecule (CD5) were targeted by 4 drugs. ST6 beta-galactoside alpha-2,6-sialyltransferase 1 (ST6GAL1), DNA damage regulated autophagy modulator 1 (DRAM1) and ADAM metallopeptidase domain 9 (ADAM9) were targeted by 1 drug.

### Verification of key lncRNAs

**FIGURE 9:**
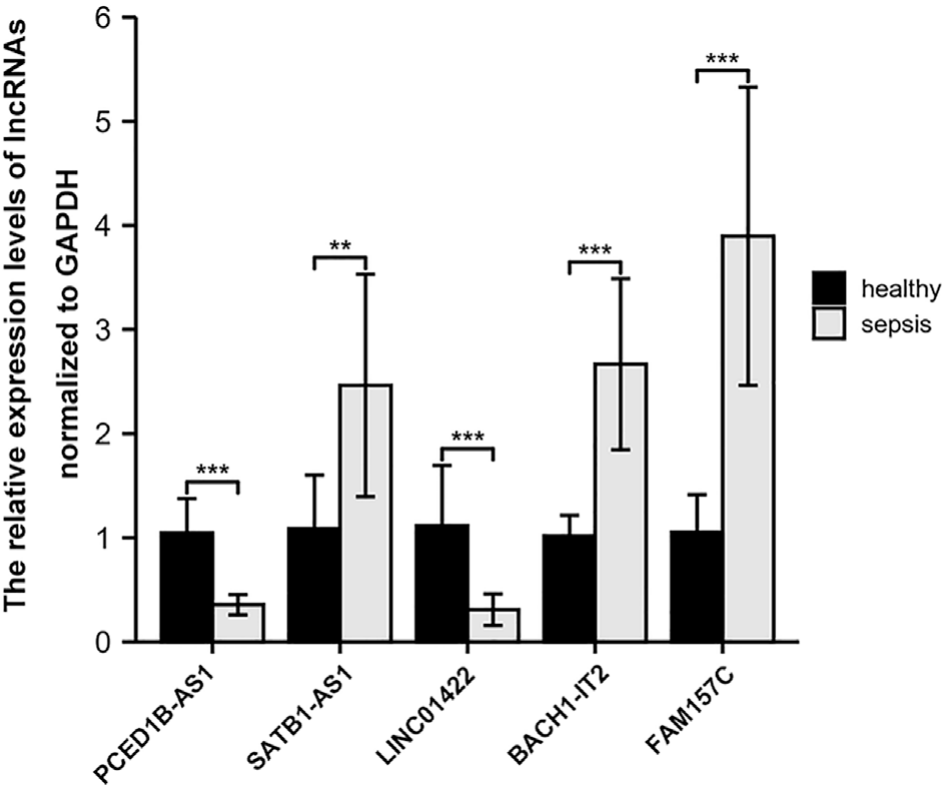
Comparison of the relative expression of lncRNAs between sepsis patients and healthy control subjects. ∗∗, *p* < 0.001; ∗∗∗,*p* < 0.001.

We chose nine blood samples from sepsis patients and nine blood samples from healthy control subjects for qRT-PCR molecular validation to ensure the authenticity of the major lncRNAs we found. The results showed that the relative expression of five lncRNAs were significantly different (*p* < 0.05) between sepsis patients and healthy control subjects (Figure 9).

## Discussion

Sepsis is one of the most serious medical diseases nowadays. Sepsis and severe sepsis account for 30% and 37% of patients in European intensive care units, respectively (Vincent et al., 2006). Gaieski et al. (Gaieski et al., 2013) point out that after heart disease and cancer, severe sepsis is the third leading cause of mortality in the United States. However, the paucity of biomarkers for the diagnosis of sepsis provides a significant hurdle for the early and accurate clinical diagnosis of sepsis. High-throughput biotechnology was developed lately and is now frequently employed in fundamental research (Layton et al., 2019). From a genetic standpoint, it is possible to demonstrate hundreds of genetic differences in illness progression, which may give a biological basis for the early and accurate diagnosis of sepsis (van der Poll et al., 2017). The hub genes of sepsis were obtained through comprehensive analysis and the ceRNA network was constructed, which laid the foundation for future research into the mechanism of sepsis progression.

In the present study, 57 DElncRNAs (32 downregulated and 25 upregulated), 6 DEmiRNAs (3 downregulated and 3 upregulated) and 317 DEmRNAs (128 downregulated and 189 upregulated) were obtained from 7 different datasets in the GEO database. Seven mRNAs (ALPL, ADM, UGCG, CD5, ST6GAL1, DRAM1 and ADAM9) were predicted to interact with drugs among twenty-eight mRNAs in the network. Both PPI and lncRNA-miRNA-mRNA networks confirmed that ETS proto-oncogene 1 (ETS1), C-C motif chemokine receptor 7 (CCR7) and CD5 are key genes of the ceRNA network in sepsis. The Ets family is made up of proteins that share a DNA binding domain (winged helix-turn-helix motif), which enables Ets proteins to interact with GGAA/T-containing DNA elements and play a crucial role in mammalian immune regulation (Wasylyk et al., 1993). ETS1, a member of the family, interacts with AP-1 and NF-B, among other transcription factors, and has a role in the regulation of Tumor necrosis factors, integrins, extracellular proteases, and genes involved in the survival of inflammatory cells (Barthel et al., 2003; Chung et al., 2005). Ryter et al. (Ryter et al., 2002) found that ETS1 is an important regulator of human natural killer (NK) cell development and terminal differentiation. Ruan et al. (Ruan et al., 2019) found that ETS1 plays an important role in the establishment of intercellular crosstalk and contributes to the commitment of cardiac lineage in pluripotent state. Heme oxygenase (HO)-1 is a cytoprotective enzyme that also has anti-inflammatory properties (Ryter et al., 2002). It has been found that EST1 can induce the activity of HO-1 promoter, drive the expression of HO-1 and prevent excessive inflammatory reaction and oxidative tissue damage during endotoxemia (Chung et al., 2005).

The protein encoded by CCR7 belongs to the G protein coupled receptor family, which activates B and T cells and is found in many lymphoid tissues. It has been demonstrated that it can inhibit memory T cell migration to inflammatory tissue while also promoting dendritic cell maturation (Bill et al., 2022). In lymph nodes, the signal mediated by this receptor regulates the homeostasis of T cells and might possibly be involved in T cell activation and polarization, as well as the pathogenesis of chronic inflammation (Bill et al., 2022). Flores-Mejía et al. (Flores-Mejía et al., 2019) examined blood samples from sepsis patients and healthy volunteers and found that CCR7 was overexpressed in NK cells. Yang et al. (Yang et al., 2022) found that the antigen-presenting cell related marker CCR7 on fresh γδ T cells was considerably greater in sepsis patients compared to the control group. In addition, Almansa et al. (Almansa et al., 2015) believe that quantitative analysis of the expression level of CCR7 and other genes is helpful to evaluate the disease severity and immunological changes of sepsis. Yin et al. (Yin et al., 2021) found that astaxanthin can downregulate the expression of CCR7 to avoid the immune dysfunction of dendritic cells, which provides a novel approach for the potential treatment of sepsis. Therefore, we speculate that CCR7 has a critical role in the diagnosis and treatment of sepsis.

The CD5 gene produces a protein that belongs to the scavenger receptor cysteine-rich (SRCR) class and functions as a T cell, B1Mura cell, and B-CLL receptor (Burgueño-Bucio et al., 2019). This receptor has been reported to be a positive and negative regulator of T cell receptor (TCR) signal transduction, as well as a negative regulator of B cell receptor (BCR) signal transduction (Soldevila et al., 2011). Recent studies have shown that CD5 promotes T cell survival by preventing activation-induced cell death in autoreactive T cells, promotes peripheral regulatory T cell induction from the start, regulates Th17 and Th2 differentiation, and modulates immune response *via* regulating dendritic cell function (Burgueño-Bucio et al., 2019). Because CD5 receptor is an immune checkpoint regulator, it can be exploited as an immunological intervention target in a variety of diseases, including cancer, autoimmune disease, and infection (Freitas et al., 2018). Vera et al. (Vera et al., 2009) found that CD5 lymphocyte receptors can detect the presence of conservative fungal components and support the therapeutic vaule of soluble CD5 forms in the treatment of fungal sepsis. Therefore, CD5 is predicted to become a biomarker and therapeutic target of sepsis.

Transcription factor enrichment analysis, GO analysis and KEGG analysis were performed on 317 DEmRNAs. The functions of transcription factor targets mainly include transcription, immune response and morphological changes of animal organs, which indicates that sepsis is a systemic immune response caused by pathogens, microorganisms or endotoxins, and severe patients have multiple organ failure or even death (Singer et al., 2016). GO analysis revealed that in BP, it was primarily enriched in neutrophil activation, T cell activation and lymphocyte differentiation. Overwhelming evidence suggests that neutrophil activation directly associated to the emergence and progression of sepsis (Ren et al., 2020). Recent evidence suggests that the function and differentiation of T lymphocytes, including the increased differentiation from Th1 (inflammation) to Th2 (anti-inflammation) and Treg cells, are related to the development of immunosuppression during the late stages of sepsis (Mori et al., 2015). Among CC, it is mainly enriched in specific granule and plasma membrane. Maitra et al. (Maitra et al., 2010) pointed out that matrix metalloproteinase-9, which is a crucial effector in acute inflammatory disorders like sepsis, is deposited in the tertiary granules of polymorphonuclear leukocytes. Johar et al. (Rahman et al., 2021) showed that the changes of cell permeability, gas exchange, cytokine migration and protein transport caused by sepsis were closely related to the plasma membrane. In MF, it is mainly enriched in transferase activity and protein serine/threonine kinase activity. Some studies have pointed out that in sepsis patients, glutathione S-transferase A1-1 can be an early indicator of liver dysfunction (Yan et al., 2014). Urinary glutathione S-transferase can be a good early indicator of renal dysfunction in intensive care patients with sepsis (Walshe et al., 2009). Previous research has indicated that serine/threonine kinases play a significant role in sepsis-induced inflammation (Packiriswamy et al., 2016). KEGG pathway analysis showed that it was primarily enriched in fluid shear stress and atherosclerosis pathway, hematopoietic lineage pathway and Th cell differentiation related pathway. No studies have reported the direct relationship between fluid shear stress and atherosclerosis pathway and sepsis, but increasingly evidence show that this pathway has a strong link to vascular oxidative stress, inflammation and atherosclerosis (Jen et al., 2013). Muranski et al. (Muranski and Restifo, 2013) have found that regulating the differentiation pathway of Th17 cells can regulate the expression of interleukin-17 and prevent excessive inflammation, which is very important for the protection of immunity and host cells. Interestingly, allergies can be caused by inappropriate growth of human Th2 cells, while autoimmunity can be caused by an excessive Th1 response, so the balance between Th1/Th2 subsets is regulated through Th1 and Th2 cell differentiation pathways, thus playing an important role in the treatment of sepsis (Amsen et al., 2009). These biological processes discovered by GSEA are obviously similar to the above results. All of the information presented above suggest that our conclusions are quite trustworth and that these DEmRNAs play a key role in sepsis.

During the previous several decades, including lncRNAs and miRNAs, they have attracted more and more attention because of their role in physiological and pathological responses. At present, more and more researches have been conducted to give evidence to support the ceRNA hypothesis that lncRNAs carrying MREs can competitively combine with some miRNAs, thus, at the post-transcriptional stage, controlling miRNA-mediated downstream target gene silencing (Khorkova et al., 2015). For example, Zhang et al. (Zhang et al., 2020) have demonstrated that lncRNA TCONS_00016233 aggravates septic acute renal injury induced by LPS by binding to miR-22-3p and preventing the down-regulation of miR-22-3p-mediated apoptosis inducing factor mitochondria associated 1 (AIFM1); Wang et al. (Wang et al., 2021) found that lncRNA-LUCAT1 can regulate the expression of ROCK1 in H9C2 cells induced by LPS by secreting miR-642a. Knockout of lncRNALUCA T1 can inhibit myocardial injury in sepsis induced by LPS; An et al. (An et al., 2021) believe that LncRNA ZFAS1, as the ceRNA of miR-138-5p, up-regulates the expression of SESN2, thus improving cardiomyocyte scorch death induced by sepsis. Therefore, it is of great significance to investigate the role and regulation mechanism of lncRNAs as ceRNAs in sepsis, as well as their potential role in diagnostics.

In this research, DEmiRNAs was used to predict the combination of lncRNAs and mRNAs of miRNAs to construct a ceRNA network. There were five lncRNAs in the network, including PCED1B-AS1, SATB1-AS1 and LINC01422 down-regulated, BACH1-IT2 and FAM157C up-regulated, but what role these lncRNAs in the progression of sepsis is not clear. It is reported that there is a certain relationship between PCED1B-AS1 and immune cells. Fan et al. (Fan et al., 2021) found that PCED1B-AS1 overexpression in hepatocellular carcinoma cells has been shown to impair the activity of co-cultured T cells and cause immunosuppression; In addition, Li et al. (Li et al., 2019) demonstrated that by directly binding to miR155, PCED1B-AS1 controls macrophage apoptosis and autophagy. Acute myeloid leukemia (AML) is an invasive hematopoietic tumor. Zhou et al. (Zhou et al., 2021a) found that inhibition of SATB1-AS1 can up-regulate miR-580 and down-regulate OAS2, thus increasing the sensitivity of AML cells. The rest of lncRNAs have not been reported before, and we are attempting to clarify their molecular mechanism in the occurrence and progression of sepsis using ceRNA hypothesis. We hypothesize that BACH1-IT2 and FAM157C may influence target gene expression in sepsis through competitive binding to has-miR-3188. To support our prediction, miR-3188 was shown to be downregulated in atherosclerotic patients, which significantly promoted macrophage damage by reducing cell viability, inducing apoptosis and increasing the production of inflammatory cytokines (including IL-1 β, IL-6, MCP-1 and TNF- α) (Li et al., 2017). Furthermore, Zhou et al. (Zhou et al., 2021b) found that miR-3188 can promote cell cycle by up-regulating cyclin cyclind1 and down-regulating p21 protein expression in polycystic ovary syndrome, resulting in abnormal proliferation of granulosa cells. For lncRNA SATB1-AS1-has-miR-4286-mRNA network, studies have shown that has-miR-4286 can improve vascular endothelial cell injury by inhibiting transforming growth factor-β1 (TGF-β1) and reducing apoptosis and inflammation (He et al., 2020). We speculate that PCED1B-AS1 may competitively combine with has-miR-7977 to change the expression of downstream sepsis-related mRNAs. Fichna et al. (Fichna et al., 2021) suggested that miR-7977 is increased in CD4+T cells of autoimmune Eddie’s disease patients and plays a crucial function in autoimmune diseases; Horiguchi et al. (Horiguchi et al., 2016) found that miR-7977 can reduce the hematopoietic support ability of bone marrow CD34^+^ cells. Our results show that LINC01422 can combine not only with has-miR-7977, but also competitively with has-miR-197-3p. Qiao et al. (Qiao et al., 2021) demonstrated that miR-197-3p attenuates apoptosis, inflammation and oxidation of epithelial cells. Similarly, Akkaya-Ulum et al. (Akkaya-Ulum et al., 2021) believe that miR-197-3p combines with the interleukin-1β receptor type I (IL1R1) gene and plays an anti-inflammatory role in monocytes and macrophages. Finally, we utilized qRT-PCR to confirm the expression levels of five important lncRNAs, and all of them were substantially different between sepsis patients and healthy control participants. Future research should focus on the precise function and mechanism of these lncRNAs in the occurrence and progression of sepsis.

Although our current findings have good clinical implications and may serve as a foundation for future study into the mechanism of sepsis, there are certain limitations to be aware of. First, our sample size is relatively small, so future study should investigate a bigger sample size to confirm the veracity of our findings. Secondly, the particular mechanism of lncRNA-miRNA-mRNA network of sepsis has to be researched further for *in vivo* and *in vitro* confirmation.

## Conclusion

In conclusion, we constructed a sepsis-specific ceRNA network to help further understand the relationship between lncRNAs, miRNAs, and mRNAs, and found that five lncRNAs were closely related to sepsis. Moreover, these discoveries will contribute to a better understanding of the pathogenesis and molecular mechanism of sepsis. We believe that our research will help to the progression of new molecular targets that will enable the early diagnosis and targeted treatment of sepsis patients.

## Data Availability

The datasets presented in this study can be found in online repositories. The names of the repository/repositories and accession number(s) can be found in the article/Supplementary Material.

## Abbreviations

GEO: gene expression omnibus; GO: gene ontology; KEGG: Kyoto encyclopedia of genes and genomes; GSEA: gene set enrichment analysis; ceRNA: competitive endogenous RNA; PPI: protein–protein interaction; lncRNA: long non-coding RNA; miRNA: microRNAs; mRNA: messenger RNA; qRT-PCR: quantitative real-time polymerase chain reaction.
