# Supplementary figures and images for: Construction of a potentially functional lncRNA-miRNA-mRNA network in sepsis by bioinformatics analysis
Source: Front Genet. 2022 Nov 15;13:1031589. doi: 10.3389/fgene.2022.1031589 (PMC9707798; doi:10.3389/fgene.2022.1031589)

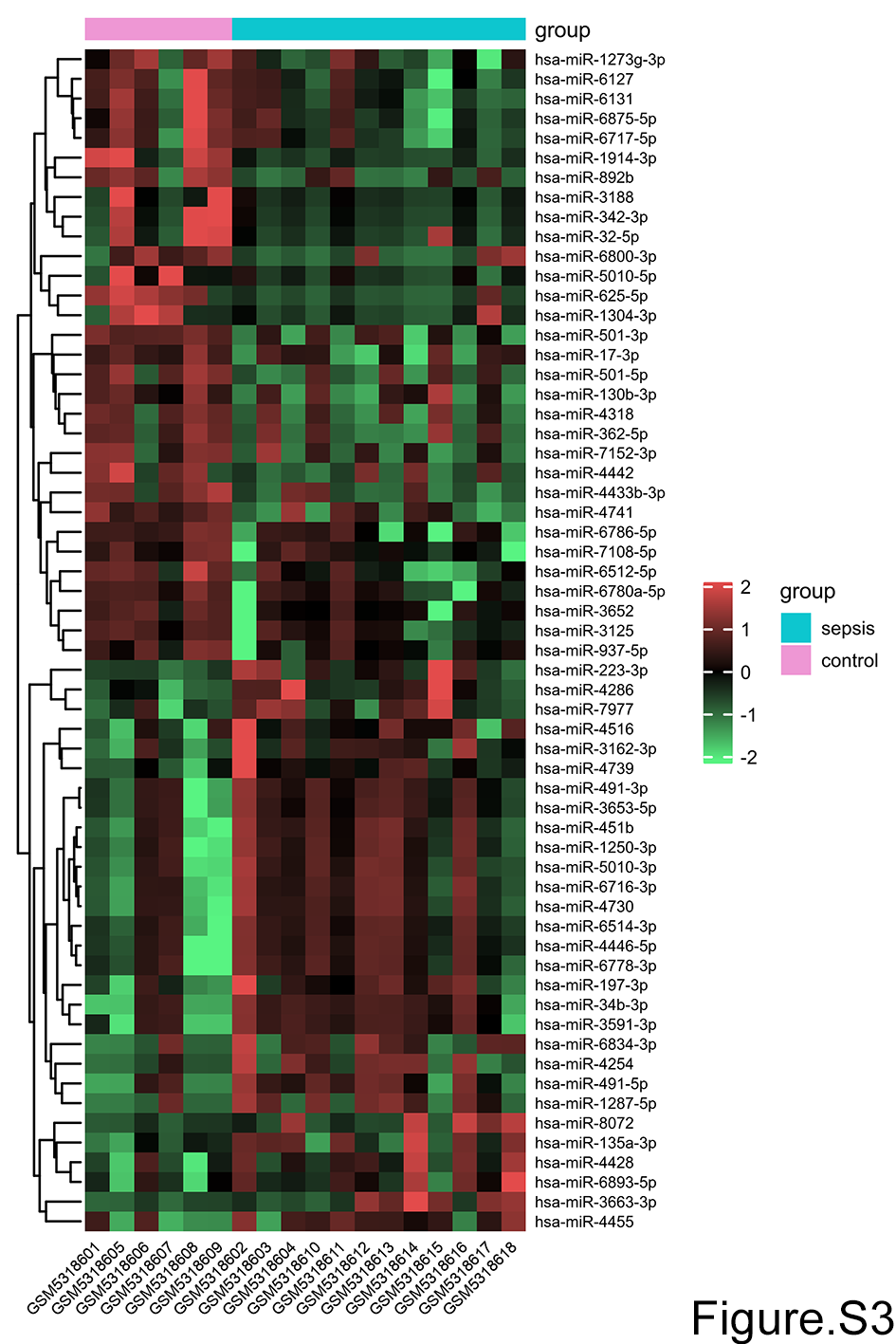

Supplement: Supplementary file 1 [file Image3.TIFF]

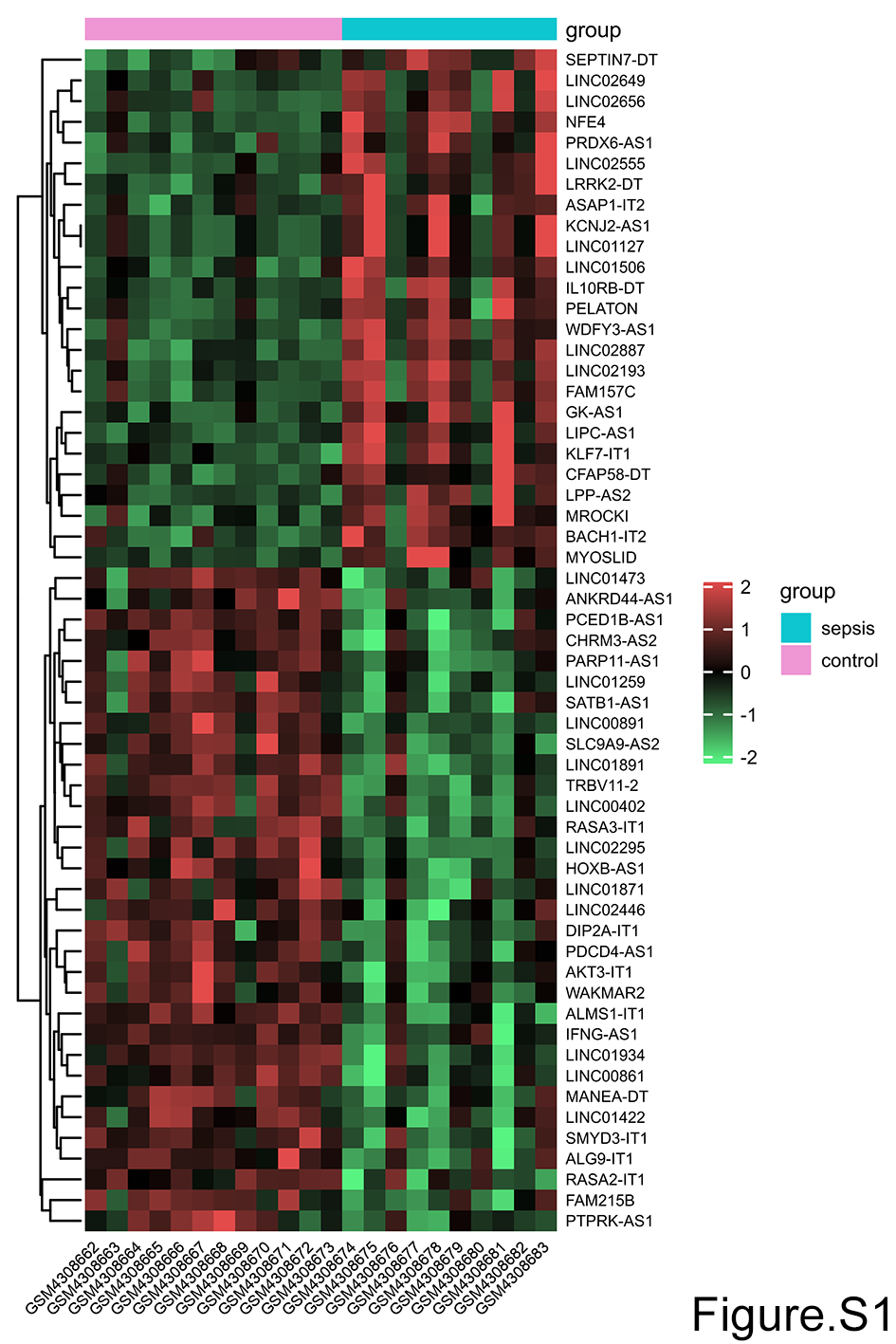

Supplement: Supplementary file 2 [file Image1.TIFF]

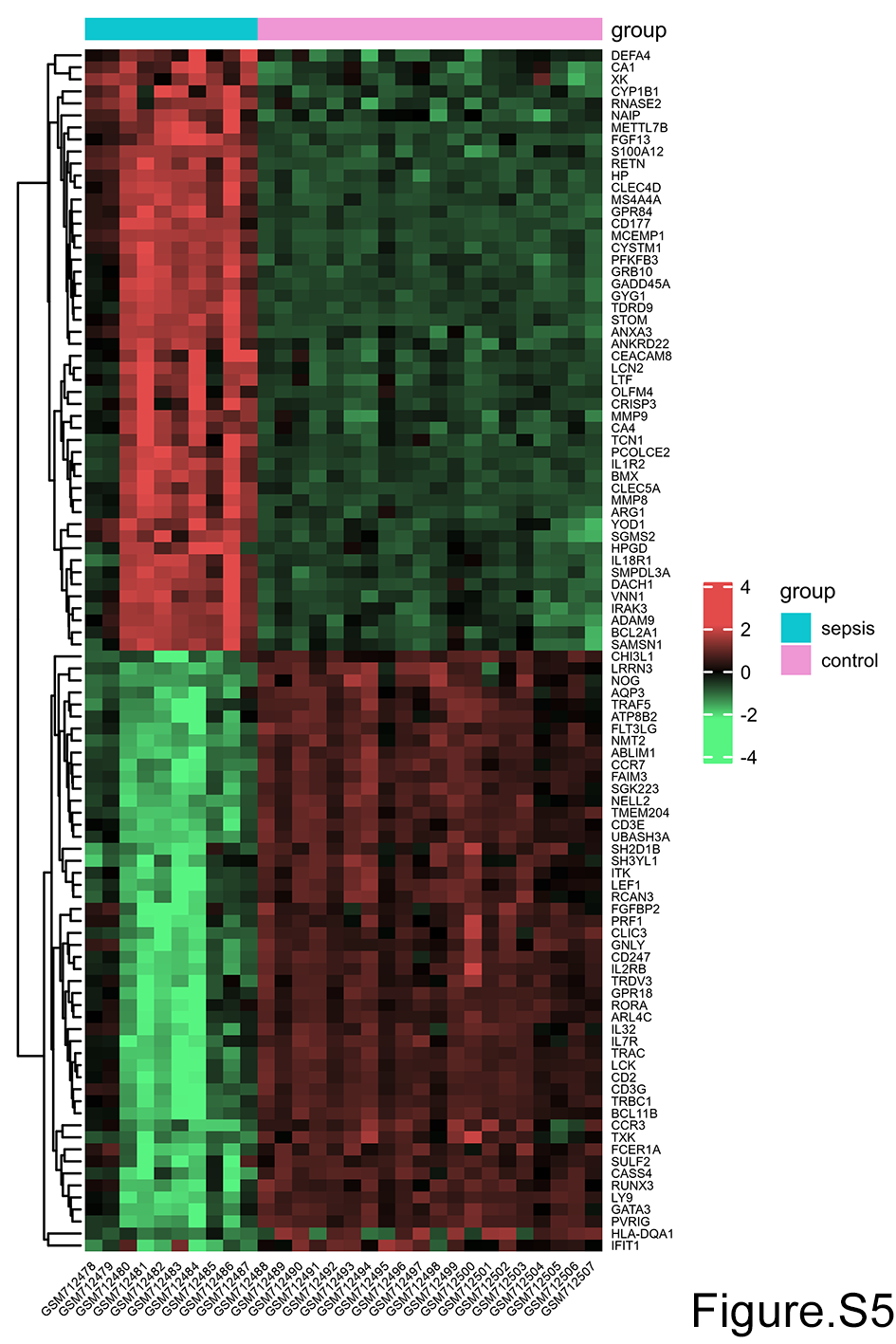

Supplement: Supplementary file 4 [file Image5.TIFF]

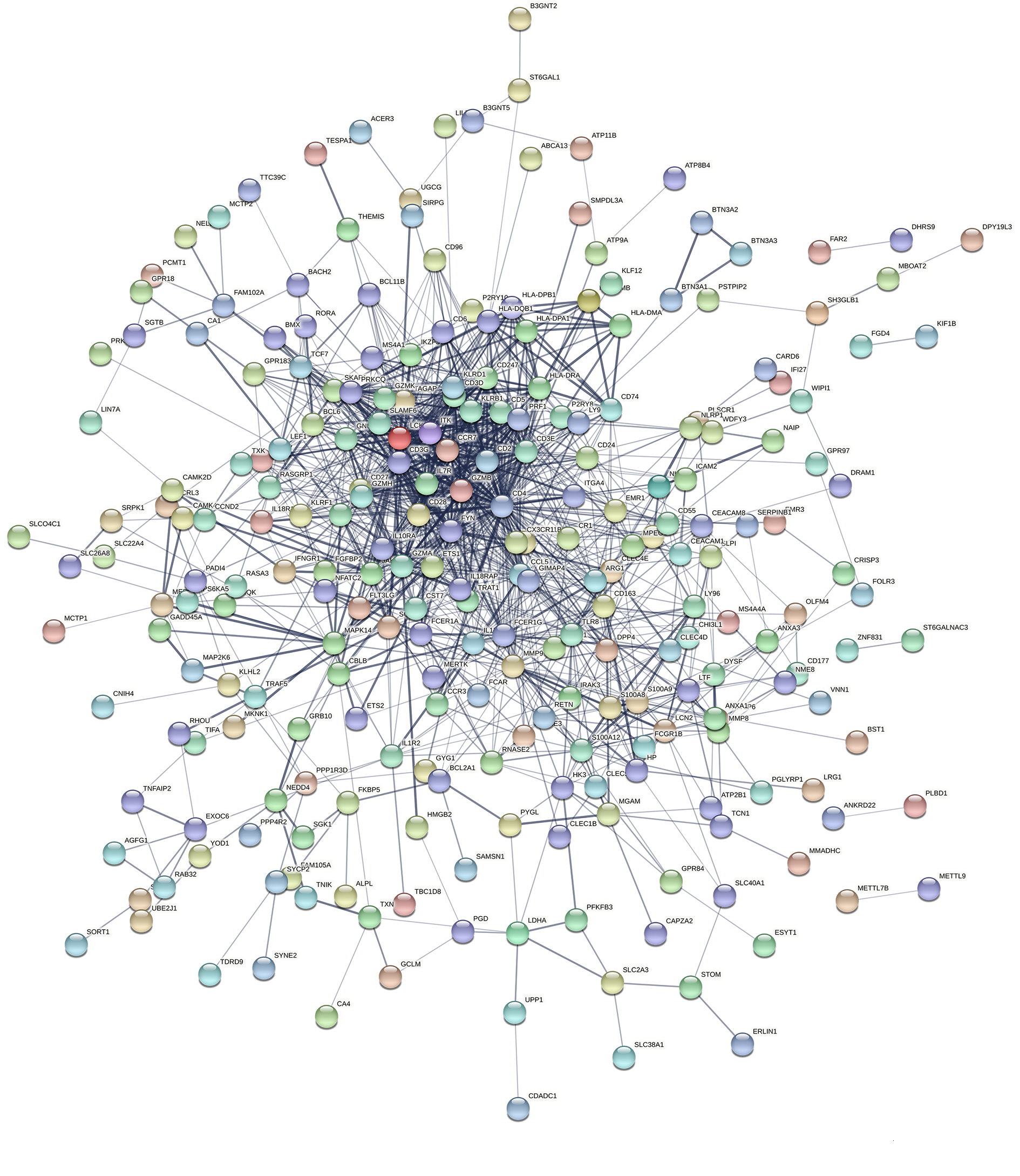

Supplement: Supplementary file 5 [file Image8.tif]

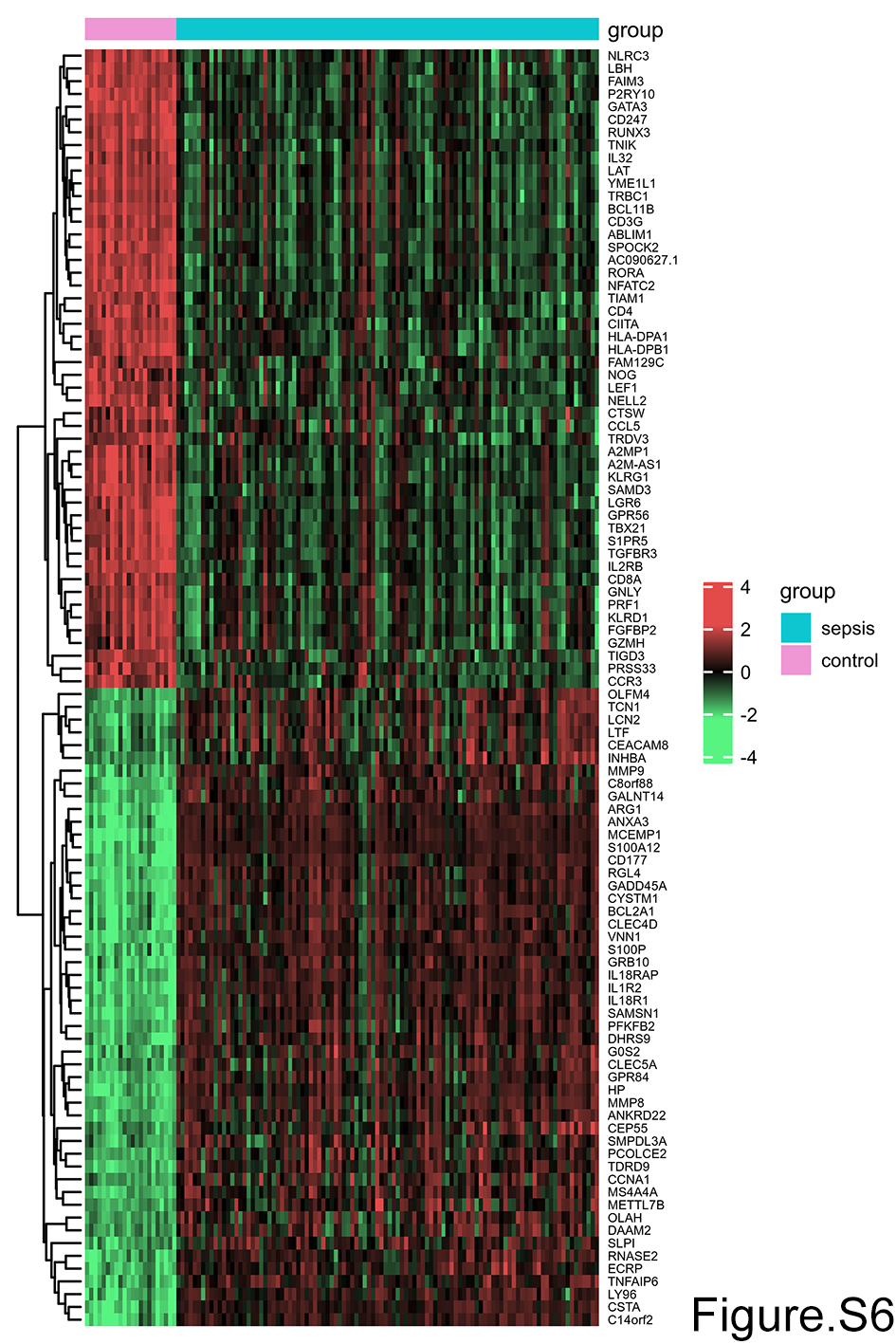

Supplement: Supplementary file 6 [file Image6.TIFF]

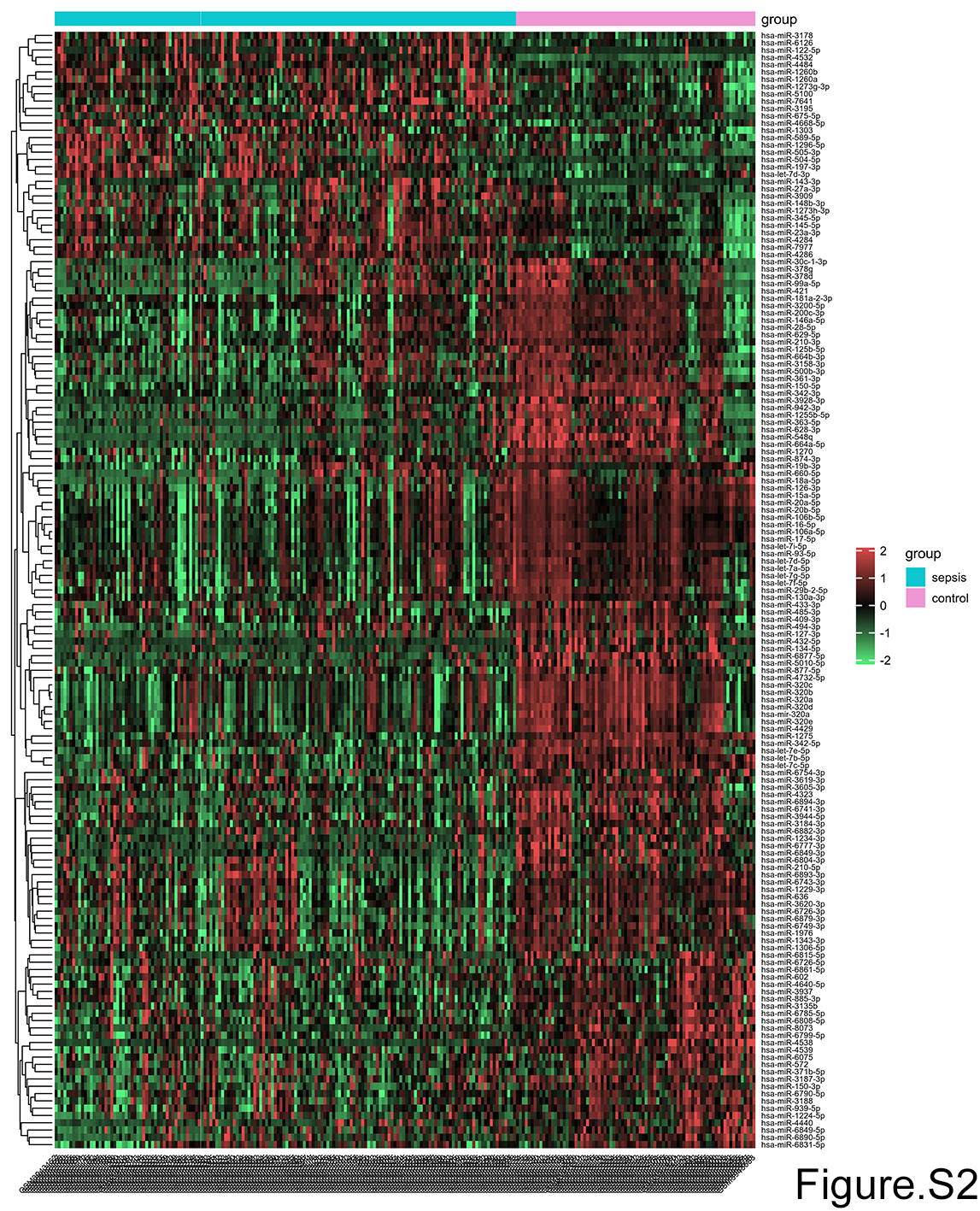

Supplement: Supplementary file 7 [file Image2.TIFF]

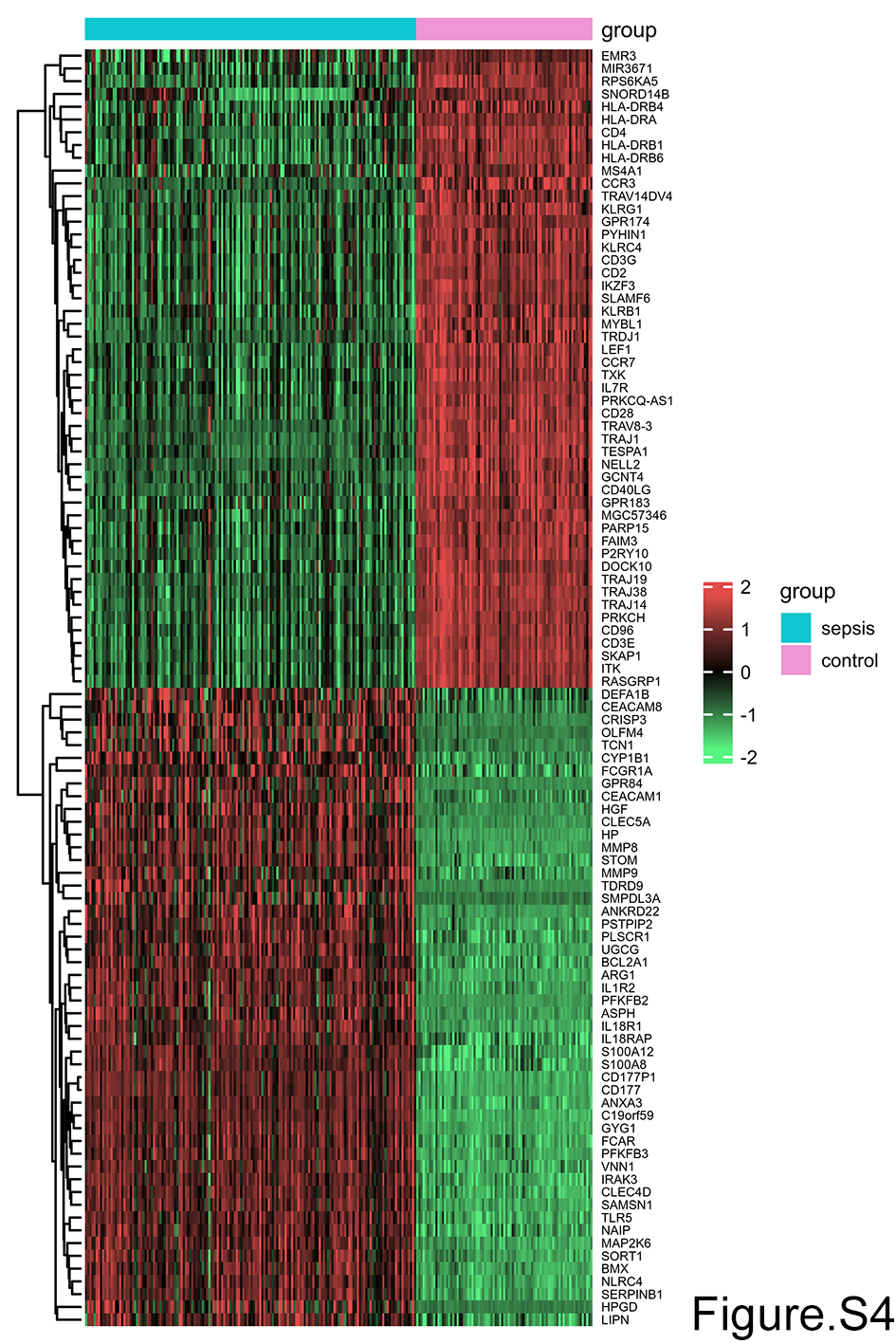

Supplement: Supplementary file 8 [file Image4.TIFF]

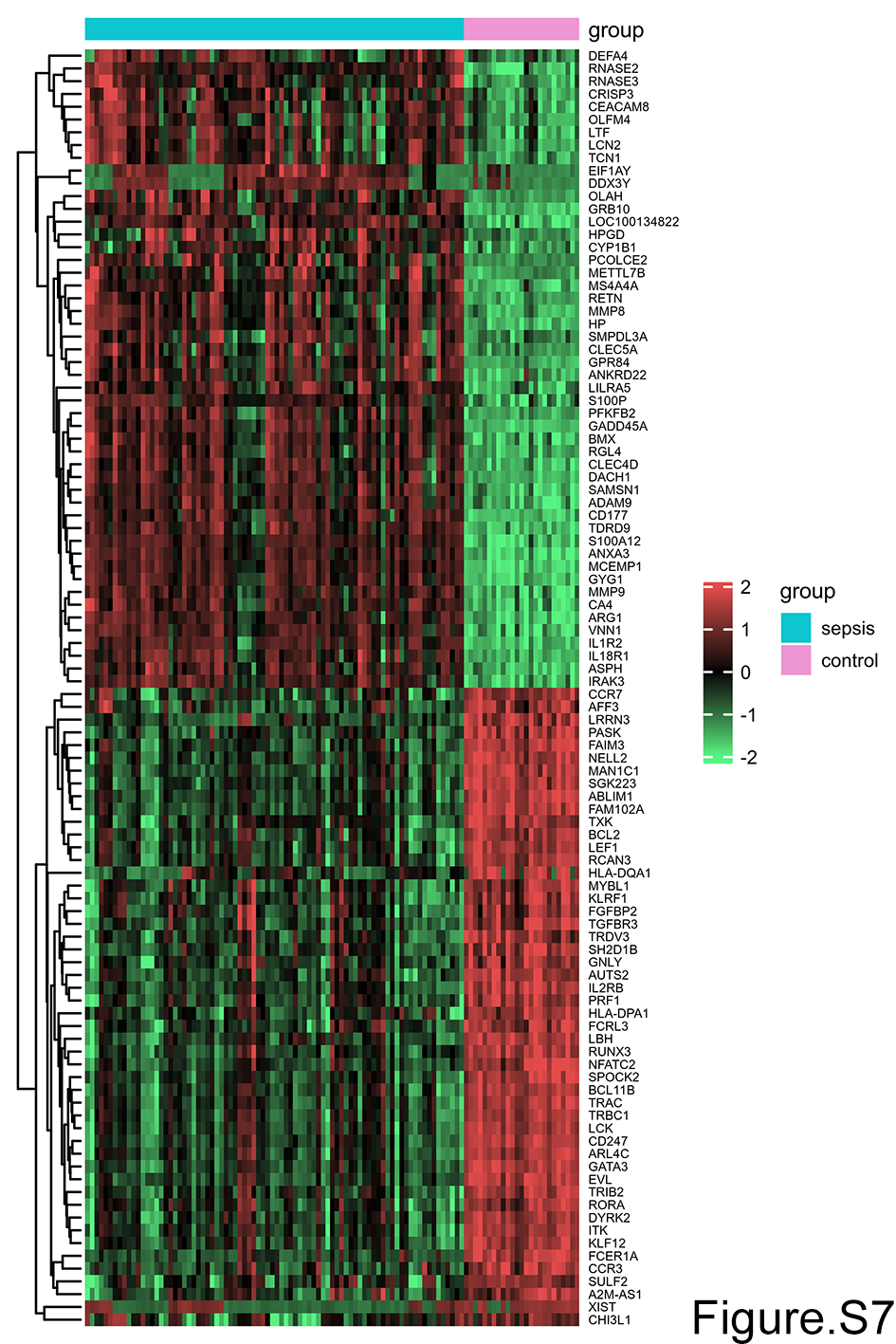

Supplement: Supplementary file 9 [file Image7.TIFF]
