# Supplementary material for: Construction of a potentially functional lncRNA-miRNA-mRNA network in sepsis by bioinformatics analysis
Source: Front Genet. 2022 Nov 15;13:1031589. doi: 10.3389/fgene.2022.1031589 (PMC9707798; doi:10.3389/fgene.2022.1031589)
Supplement: Supplementary file 3 [file Table1.docx]

| **Name** | **Primer sequence (5’-3’)** | | **Size** |
| --- | --- | --- | --- |
| H-GAPDH | Forward: | CATCATCCCTGCCTCTACTGG | 259 |
|  | Reverse: | GTGGGTGTCGCTGTTGAAGTC |  |
| PCED1B-AS1 | Forward: | TAAATGAAGATGGAGAATCAGACAT | 245 |
|  | Reverse: | TGCCATAATTTAACCAACATATATC |  |
| SATB1-AS1 | Forward: | AATGAAAAGGGTGGAAGAGTAAACT | 251 |
|  | Reverse: | TGTGAAATTGATACCTTTCCATCAT |  |
| LINC01422 | Forward: | CTCCTGGTCTCAGGCACCTAG | 168 |
|  | Reverse: | TGAGTTTGAAGAGGAGCTGATG |  |
| BACH1-IT2 | Forward: | TGAACTCTTCGGTCTAGAGACAGAT | 232 |
|  | Reverse: | CATCTGACACATTAGGTCACATAGC |  |
| FAM157C | Forward: | CAACTGGATTTGCTTTTTCACC | 283 |
|  | Reverse: | GGATCTCCGAAGTTCACTGTTC |  |

Supplementary table 1. Primer information.
